# KETAMIR-2, a new molecular entity and novel ketamine analog

**DOI:** 10.3389/fphar.2025.1606976

**Published:** 2025-06-20

**Authors:** Itzchak Angel, Rita Perelroizen, Wendy Deffains, Florian W. Adraoui, Eddy Pichinuk, Erez Aminov

**Affiliations:** ^1^ Mira Pharmaceuticals Inc., Miami, FL, United States; ^2^ Pharmaseed Ltd., Ness Ziona, Israel; ^3^ Biotrial, Rennes, France

**Keywords:** ketamine, ketamir-2, anti-depressant, NMDA, neuropathic pain, hyperlocomotion

## Abstract

Ketamir-2 is a new molecular entity, and a novel ketamine analog designed to improve oral bioavailability, and offer a superior safety profile compared to existing ketamine treatments. It was found that Ketamir-2 is a low affinity NMDA receptor antagonist, that selectively binds to the PCP site. Its IC_50_ on this receptor site is ∼100 µM. Ketamine acts as an NMDA receptor antagonist (0.5–1 µM affinity) and influences opioid receptors, monoaminergic systems (such as serotonin and dopamine). The selectivity of Ketamir-2 was evaluated across a broad range of receptors, transporters, and binding sites, but no activity was detected. Ketamir-2 is a lower affinity and selective PCP-site NMDA antagonist, compared with ketamine. Upon oral administration, Ketamir-2 does not induce hyperlocomotion in mice. Thus, it is different from Ketamine, which induces marked hyperlocomotion, a behavior which is thought to mimic the psychomotor agitation and disorganized behavior seen in schizophrenia, often linked to disruptions in brain neurotransmitter systems. Ketamir-2 was also evaluated via several pharmacological tests in mice to evaluate its anti-depressive and anxiolytic effects. These tests included the Open Field test (OFT), Elevated Plus Maze (EPM), and Forced Swimming Test (FST); all of which are commonly employed behavioral assays used to evaluate the efficacy of anxiety and depression medications. While showing significant effects in these tests at variable doses, ketamine, which was used as a positive control, did not differ from vehicle treatment or showed an opposite response to Ketamir in the majority of the tests studied.

## Introduction

Ketamir-2 is a new molecular entity and novel ketamine analog designed to have an improved oral bioavailability, and offer a superior safety profile compared to existing ketamine treatments. Unlike ketamine, which acts broadly as an N-Methyl-D-aspartic acid (NMDA) receptor antagonist, influencing multiple receptor sites including opioid and monoaminergic systems (Zorumski et al., 2016), Ketamir-2 selectively targets the phencyclidine (PCP) site of the NMDA receptor with lower affinity. This selective binding reduces the risk of adverse effects, such as sedation and dissociation, commonly seen with ketamine (Orhurhu et al., 2023), while aiming to maximize therapeutic outcomes for conditions like depression, anxiety, and neuropathic pain.

The binding of ketamine to the PCP site within the NMDA receptor is crucial as it allows for noncompetitive antagonism of the receptor, effectively blocking its activity (Sharp, 1996). This binding mechanism contributes to ketamine’s analgesic and antidepressant effects, as it disrupts normal glutamatergic neurotransmission (Mion and Villevieille, 2013). Additionally, the high trapping capability of ketamine at the PCP site enhances its potency and duration of action in modulating synaptic activity. In addition to the PCP site on the NMDA receptor, ketamine also binds to the sigma-1 receptor, which is implicated in various neuroprotective and antidepressant effects (Robson et al., 2012). Furthermore, ketamine has been shown to interact with opioid receptors, particularly the mu-opioid receptor, contributing to its analgesic properties (Sarton et al., 2001). These multiple binding sites highlight ketamine’s complex pharmacological profile and its potential therapeutic applications beyond anesthesia. Ketamine and its metabolites also interact with several neurotransmitter transporters, including the serotonin transporter (SERT) (Spies et al., 2017) and norepinephrine transporter (NET) (Liebe et al., 2018). Ketamine acts as an uptake inhibitor for SERT, with reported IC50 values indicating significant inhibition of serotonin uptake, which may contribute to its antidepressant effects (Spies et al., 2017). Additionally, it has been shown to inhibit dopamine and norepinephrine uptake, further influencing neurotransmitter dynamics in the central nervous system (Hess et al., 2021).

The connection to schizophrenia arises from ketamine’s ability to disrupt normal brain signaling, particularly in regions like the prefrontal cortex, nucleus accumbens, and hippocampus, which are also implicated in schizophrenia (Abdallah et al., 2018). Research shows that ketamine increases dopamine turnover in the nucleus accumbens, a brain area involved in regulating movement and reward. This dopamine surge aligns with the dopamine hypothesis of schizophrenia, which suggests that excessive dopamine activity contributes to psychotic symptoms (Hunt et al., 2005)​. Additionally, ketamine reduces the activity of inhibitory GABAergic interneurons, leading to a disinhibition of excitatory neurons. This imbalance creates a state of neural hyperactivity, potentially mirroring the sensory and perceptual distortions seen in schizophrenia. Studies also indicate that ketamine-induced hyperlocomotion can be blocked by dopamine receptor antagonists like haloperidol, reinforcing the link between this behavior and dopaminergic mechanisms relevant to schizophrenia (Irifune et al., 1991)​. However, the effect is not solely dopamine-driven—ketamine’s impact on glutamate and other systems, like endocannabinoids and serotonin, suggests a broader disruption of neural networks (Xu, et al., 2020)​. In this respect, it was interesting to assess the effect of Ketamir-2 in the same model.

Recently, the *S*-enantiomer of Ketamine, esketamine, has shown efficacy as an adjunct therapy in treatment resistance depression receiving approval for use in combination with an oral antidepressant (Daly et al., 2018). Rat models have been extensively used to assess the antidepressant and anxiolytic effects of ketamine. These models provide valuable insights into ketamine’s rapid-acting and long-lasting effects on mood and anxiety-related behaviors. Several studies have investigated the effects of ketamine using the Open Field Test (OFT) (Fitzgerald et al., 2021), Elevated Plus Maze (EPM), and Forced Swimming Test (FST) in rodent models. The elevated plus maze (EPM) is a widely used behavioral test to assess anxiety-like behavior in rodents, and it has been employed in several studies to investigate the effects of ketamine. Acute administration of ketamine was shown to increase time spent in open arms of the EPM in both saline- and morphine-conditioned mice, indicating an anxiolytic-like effect (McKendrick et al., 2020)​. Also, Ketamine induced antidepressant-like effects in the FST at 30 min and 24 h post-administration, reducing immobility time (Parise et al., 2013)​. We have employed these models to assess the potential antidepressant and anxiolytic effects of Ketamir-2.

**FIGURE 1:**
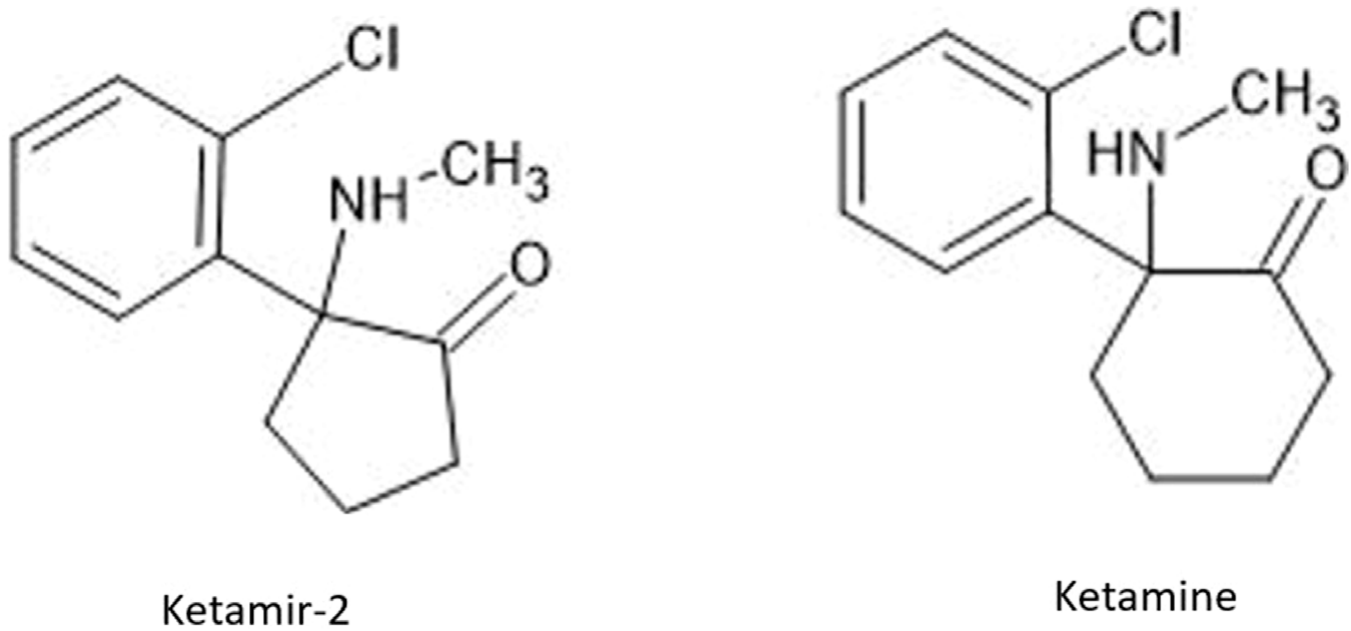
Structures of ketamir-2 and ketamine.

Ketamir-2 is a five membered ring analogue of ketamine (Figure 1). In the *in vivo* studies we have used the hemipamoate salt of Ketamir-2, also known as 1-(2-chlorophenyl)-N-methyl-2-oxocyclopentan-1-aminium 4,4′- hemi-salt of methylenebis (3-hydroxy-2-naphthoate); Ketamir pamoate; Ketamir hemipamoate; M209. Its chemical formula is C_47_H_44_Cl_2_N_2_O_8_ with a molecular weight of 835.77 g/mol. The chemical structure is provided in Figure 1. This drug is a racemic mixture, with 1:1 ratio between the R- and S- enantiomers.

## Results

### *In vitro* pharmacology: glutamate binding assays study of ketamir-2 HCl

**FIGURE 2:**
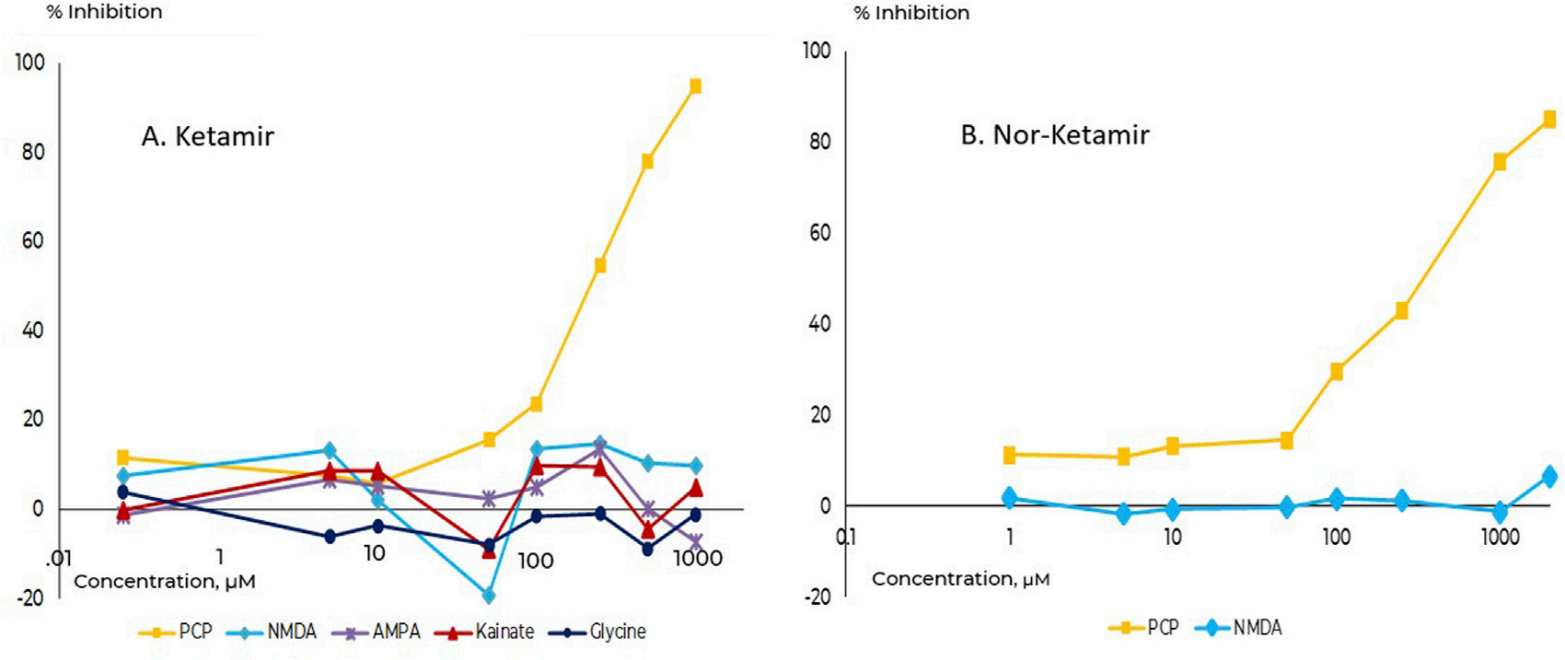
Comparative binding affinities towards the PCP-site on NMDA receptor. **(A)** Ketamir-2, **(B)** Nor-Ketamir. Inhibition of the PCP site is depicted in squares.

Ketamir-2 (in all *in vitro* studied as the HCl salt to improve solubility) was evaluated on a large array of receptors, transporters and binding sites (see below). It was found that Ketamir is a low affinity NMDA receptor antagonist, that selectively binds to the PCP- site. Its IC_50_ on this receptor site is ∼100 µM. Other sites at the Glutamate-NMDA receptor complex were also evaluated. This includes the NMDA site, the Kainate binding site, the AMPA site as well as the Glutamate non-selective ion channel. At all these sites, no significant effects were seen up to 1 mM (Figure 2).

The major metabolite of Ketamir, desmethylketamir (also called Nor-Ketamir), was evaluated under the same experimental conditions. It showed activity only towards the PCP-site with IC_50_ of ∼470 µM. Similar to Ketamir, nor-Ketamir did not show any significant activity at any of the sites studied (at 10 µM). Similarly, it did not interact with the NMDA site up to 3,000 uM. These results are also included in the *in vitro* pharmacology glutamate binding assay study of nor-Ketamir.

When this data is compared with reference Ketamine (data for the racemic mixture RS-ketamine) or other NMDA receptor antagonists (Table 1), it can be seen that Ketamir-2 and its metabolite represent a lower affinity to the NMDA receptor, compared with these compounds.

**TABLE 1:** Comparative binding affinities of Ketamir-2 and Nor-Ketamir with other NMDA receptor antagonists.

Compound	PCP site	GluN1/GluN2A NMDA
	IC_50_/Ki, µM	IC_50_/Ki, µM
Ketamir-2	100	>1,000
Nor-Ketamir	470	>3,000
Ketamine	0.006*	0.5**
PCP	0.007*	0.06**
(+) MK-801	0.006*	0.002**

*Roth et al. (2013), **Dravid et al. (2007).

### Selectivity towards other potential binding sites

The potential interaction of Ketamir-2 with other channels, binding sites and enzymes was evaluated, using a panel of several of these sites and Ketamir-HCl at 10 µM. At none of these sites Ketamir-2 had an effect above 20% (see details in the Supplement).

The sites include:

Nuclear receptors: Androgen receptors (AR) and Glucocorticoid receptor (NR3C1).

Transporters: 5HTT (SLC6A4), NET (SLC6A2) and DAT (SLC6A3).

GPCRs: Agonist and Antagonist sites: CB1/CNR1, M2/CHRM2, delta/OPRD1, kappa/OPRK1, mu/OPRM1, 5-HT1A/HTR1A, 5-HT1B/HTR1B, M3/CHRM3, alpha1A/ADRA1A, CCKA/CCKAR, H1/HRH1, M1/CHRM1, V1A/AVPR1A, A2A/ADORA2A, beta1/ADRB1, D2/DRD2, H2/HRH2, CB2/CNR2, D1/DRD1, beta2/ADRB2, ETA/EDNRA, 5-HT2A/HTR2A and 5-HT2B/HTR2B.

Enzymatic assays: COX-1, COX-2, ACHE, PDE3A, PDE4D, LCK and MAO-A.

Ion channels as agonists and antagonists: HTR3A, GABA and nAchR. As antagonists only: hERG, hKv7.1,

These results suggest, that under the experimental set up, contrary to ketamine, Ketamir-2 does not interact with a large variety of sites, transporters and channels. Ketamine has been reported to be a potent antagonist of both PCP, NR2A and NR2B sites at the NMDA receptors, with IC_50_s in the low and sub-micromolar range (Dravid et al., 2007; Roth et al., 2013; Sarton et al., 2001). In addition to this presumed primary mode of action, ketamine has multiple additional targets. For example, it binds to and activate dopamine D2 receptors in the nanomolar range, though binding affinity for the other dopamine receptor subtypes seems limited and is hardly investigated. Additionally, ketamine is also a modest inhibitor of nicotinic acetylcholine receptors (nACh), voltage-gated sodium channels and the monoamine reuptake transporters (DAT, NET and SERT) having affinities at the micromolar range (Zorumski et al., 2016)​.

### Evaluation of the acute effects of ketamir-2 on spontaneous locomotor activity in the mouse

Subanesthetic doses of ketamine induce hyperlocomotion in mice, which is considered analogous to psychotic symptoms in humans (Qin et al., 2021)​. The objective of this study was to evaluate the effects of Ketamir pamoate administration on locomotor activity on male C57BL6/J mice compared with Ketamine. Measurements of spontaneous locomotor activity by actimetry are widely used for *in vivo* safety evaluation of new drugs on the central nervous system (CNS) in rodents. This test was performed using infrared beam activity meters.

**FIGURE 3:**
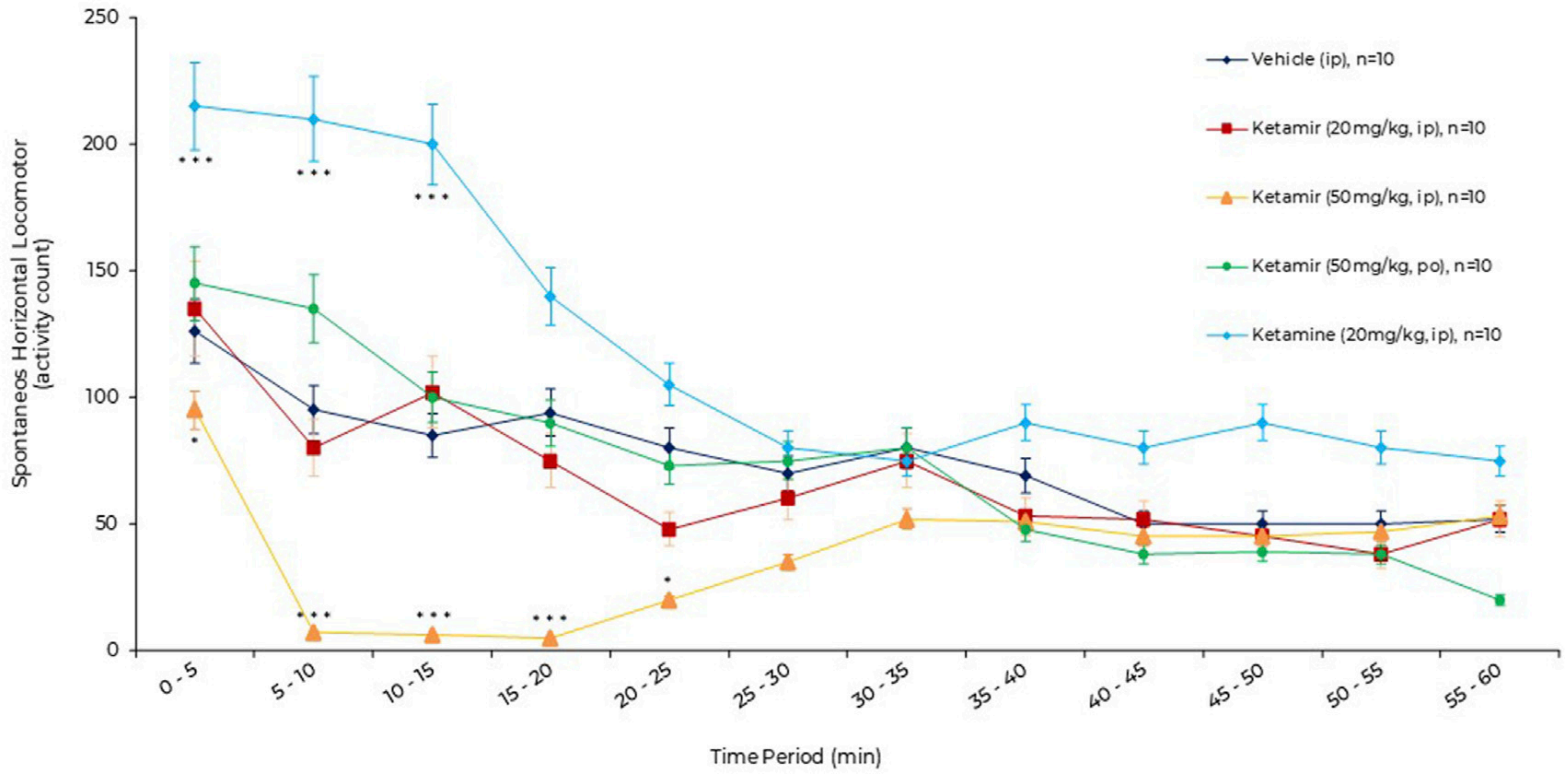
Effects of acute oral administration of Ketamir pamoate, ketamine HCl or vehicle on the spontaneous horizontal locomotor activity over a period of 1 h by 5 min intervals in male C57BL6/J mice. Results are expressed as mean ± SEM. * and ***: p < 0.05 and p < 0.001 for ketamir-2 (50 mg/kg, IP) vs. vehicle (IP) by two-way ANOVA test followed by a Dunnett’s multiple comparisons test. ***: p < 0.001 for ketamine (20 mg/kg, IP) vs. vehicle (IP) by two-way ANOVA test followed by a Sidak’s multiple comparisons test.

**FIGURE 4:**
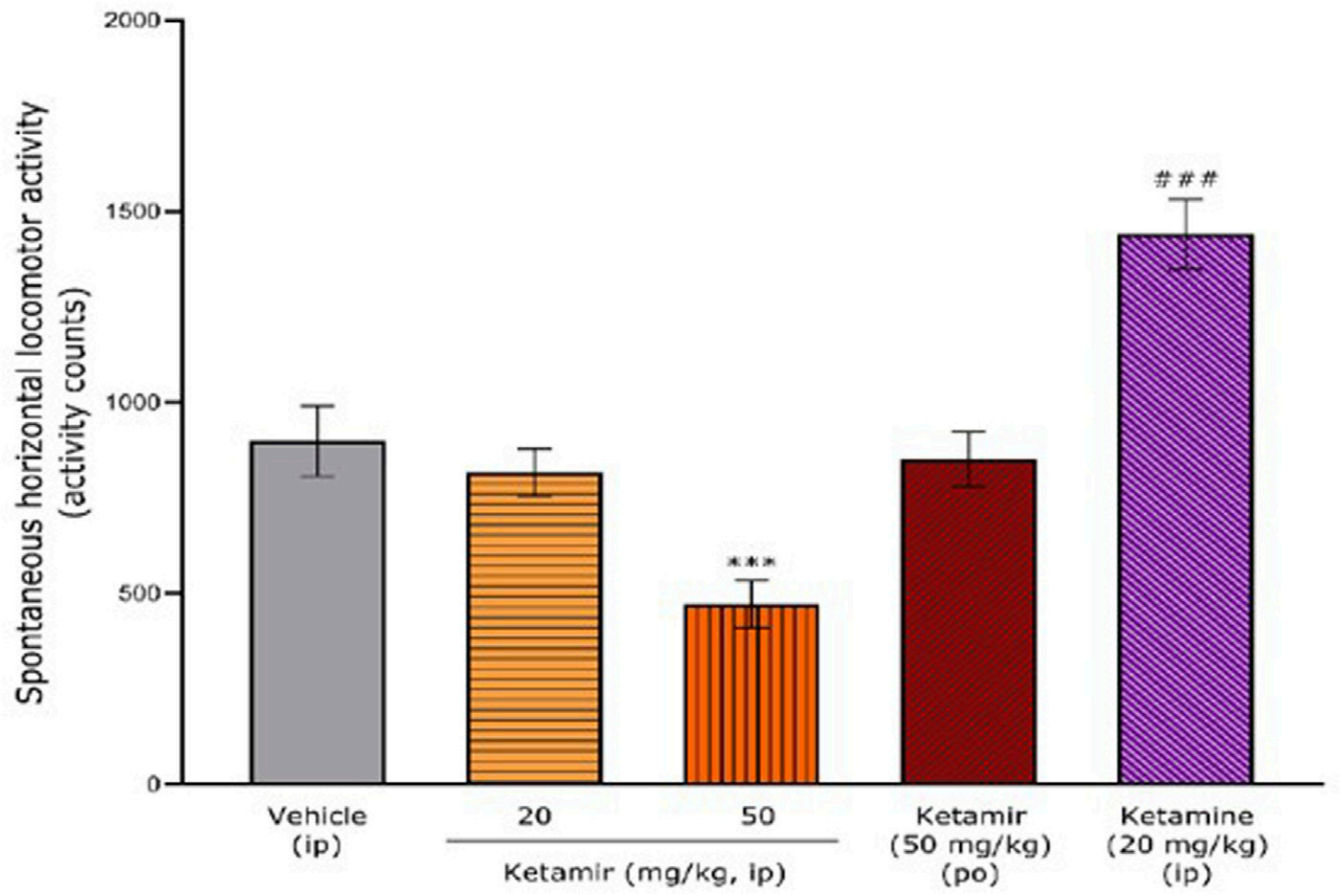
Effects of acute oral administration of Ketamir-2, ketamine or vehicle on the spontaneous horizontal locomotor activity over the entire recording period (1 h) in male C57BL6/J mice. Results are expressed as mean ± SEM. ***: p < 0.001 for Ketamir-2 (50 mg/kg, IP) vs. vehicle (IP) by one-way ANOVA test followed by a Dunnett’s multiple comparisons test. ###: p < 0.001 for ketamine (20 mg/kg, IP) vs. vehicle (IP) by two-tailed Student’s t-test for independent samples.

No clinical sign was observed. No sacrifice of animals had to be performed for humane reasons, because animals did not show any signs of permanent suffering, pain or fear as described in the OECD guide “Recognition, Assessment and Use of Clinical Signs as Humane Endpoints for Experimental Animals Used in Safety Evaluation (OECD, 2000)”. Results are presented in Figures 3 and 4 as mean ± standard error of the mean (SEM).

### Horizontal locomotor activity after ketamir, ketamine or vehicle administration

The animals were placed individually into cages positioned in an activity meter (Imetronic system) for a period of 1 h, starting just after administration. This apparatus uses infrared beams to record horizontal animal displacements. The animals had no access to food and water during the recording period.

Effects of acute oral administration of vehicle (1% DMSO/20% 2-hydroxypropyl-beta-cyclodextrin, IP) or Ketamir (20 mg/kg, IP or 50 mg/kg IP or PO) or Ketamine (20 mg/kg, IP) on the spontaneous horizontal locomotor activity (activity counts) over a period of 1 h by 5 min interval using the locomotor activity test in male C57BL6/J mice are described in Figure 3.

After vehicle administration, spontaneous horizontal locomotor activity was initially intense (0–5 min, 127 ± 7 activity counts, exploration phase), then gradually decreased (habituation phase) until 50–55 min (51 ± 11 activity counts). After administration of Ketamir 20 mg/kg IP and Ketamir 50 mg/kg PO, the horizontal locomotor activity profiles were broadly similar to that after vehicle administration.

After administration of Ketamir 50 mg/kg IP, the horizontal locomotor activity gradually decreased. The horizontal locomotor activity profile was significantly lower than after vehicle administration from 0–5 min (96 ± 9 versus 127 ± 7 activity counts, respectively, Dunnett’s multiple comparisons test, p = 0.0336) to 20–25 min (23 ± 9 versus 82 ± 17 activity counts, respectively, Dunnett’s multiple comparisons test, p = 0.0234). Thereafter, horizontal locomotor activity increased, returning to the level of the vehicle profile between 35–40 min and 55–60 min.

After administration of Ketamine 20 mg/kg IP, the horizontal locomotor activity gradually increased. The horizontal locomotor activity profile was significantly higher than after vehicle administration from 0–5 min (216 ± 14 versus 127 ± 7 activity counts, respectively, Dunnett’s multiple comparisons test, p = 0.0007) to 10–15 min (200 ± 18 versus 84 ± 13 activity counts, respectively, Dunnett’s multiple comparisons test, p = 0.0009). Thereafter, horizontal locomotor activity decreased to the level of the vehicle profile between 25–30 min and 55–60 min.

Global effects of acute oral administration of vehicle (1% DMSO/20% 2-hydroxypropyl-beta-cyclodextrin, IP) or Ketamir (20 mg/kg, IP or 50 mg/kg IP or PO) or Ketamine (20 mg/kg, IP) on the spontaneous horizontal locomotor activity (activity counts) over a period of 1 h using the locomotor activity test in male C57BL6/J mice are described in Figure.

The mean ± SEM spontaneous horizontal locomotor activity after vehicle administration was 899 ± 92 activity counts over the total period of 1 h.

After administration of Ketamir 20 mg/kg IP and Ketamir 50 mg/kg PO, mean ± SEM horizontal locomotor activity 0–60 min period were very close to that observed after vehicle administration (816 ± 63 and 852 ± 72 activity counts, respectively). After administration of Ketamir 50 mg/kg IP, mean ± SEM horizontal locomotor activity 0–60 min period significantly decreased compared with vehicle group (472 ± 61 activity counts; Dunnett’s multiple comparisons test p = 0.0006 for Ketamir 50 mg/kg IP *versus* vehicle).

After administration of Ketamine 20 mg/kg IP, mean ± SEM horizontal locomotor activity 0–60 min period significantly increased compared with vehicle group (1,439 ± 92 activity counts; Two-tailed Student’s t-test for independent samples p = 0.0006 for Ketamine 20 mg/kg IP *versus* vehicle). See Figure 4.

### Antidepressant and anxiolytic studies in mice

The objective of this study was to determine the anti-depressive and anxiety dose-response activity of Ketamir pamoate using the EPM, open field and FST tests in male mice. In these studies, Ketamir-2 or ketamine were administered once orally at the indicated dose and the animals were recorded for the indicated time period 20 min after treatment. A total of 40 male mice were utilized and randomly divided into five groups of 8 animals in each group. The number of groups and the total number of animals was based on previous studies demonstrating that this is the minimum number of animals sufficient to obtain indicative/significant information for each tested timepoint. The animals were observed for toxic/adverse symptoms: before each dosing, and before termination. No abnormal clinical signs were observed in any animal. There was no difference in the weight of animals during the study or due to treatment.

### Open field test

**FIGURE 5:**
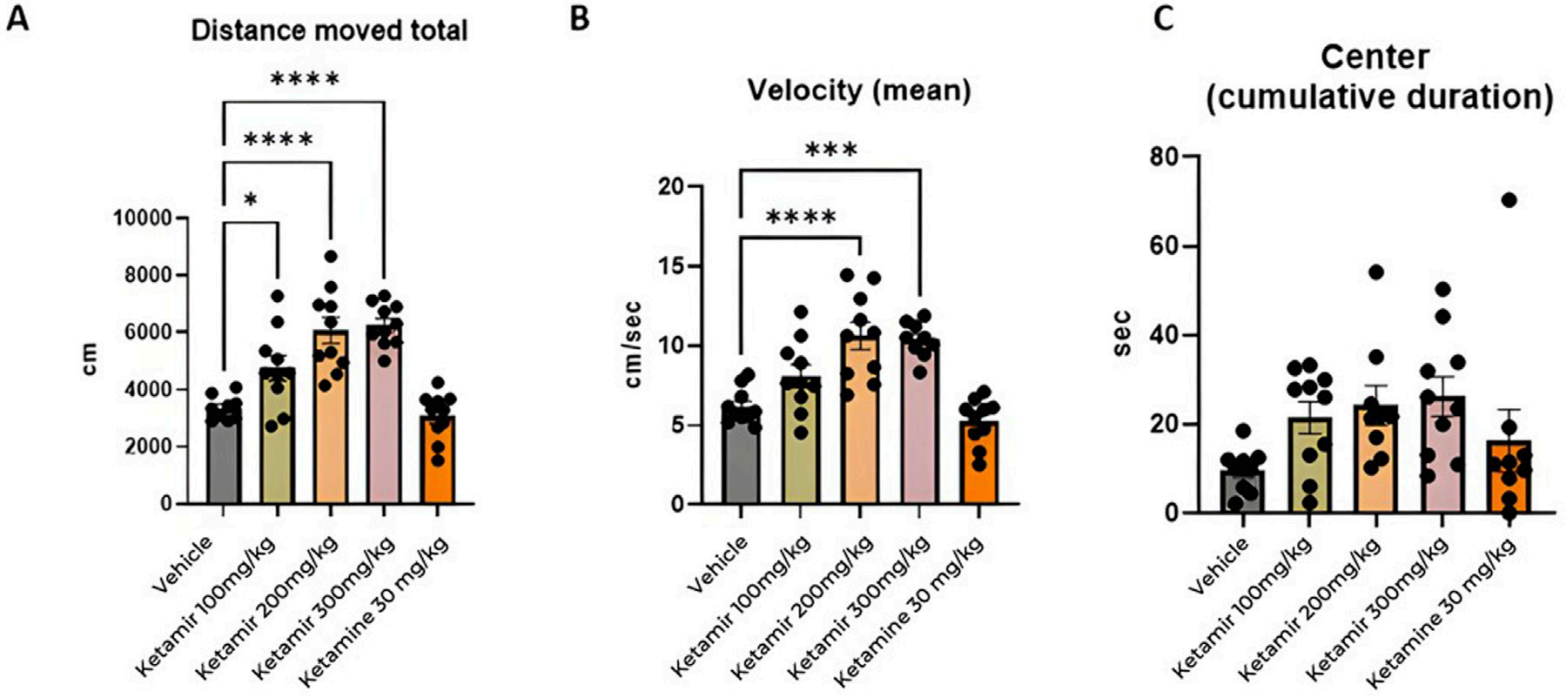
Open field parameters. In each group 8 mice were evaluated, **(A)** Total distance moved (cm); **(B)** mean velocity (cm/seconds) and **(C)** cumulative time spent in center (seconds). One-Way ANOVA compared to vehicle control: *p < 0.05 and ***p < 0.001, ****p < 0.0001.

Open field was performed on Day 1 of the study. Twenty minutes after treatment, mice were placed in the center of a chamber and allowed to freely explore the chamber for the duration of the test session (10 min). Mice were tested and recorded using a video camera installed on the ceiling of the behavioral room and connected to a computer to record images. To process and analyze behavioral parameters detected in videos, the computer software Ethovision was used. The graphical representation of the data obtained is shown in Figure 5.

#### Distance moved

Mice treated with Vehicle (group 1M) exhibited significantly less travel distance during the open field study, compared to all groups treated with Ketamir-2 (groups 2M, 3M and 4M). Data presented in Figure 5A.

#### Velocity

Mice treated with Vehicle (group 1M) traveled slower compared to groups treated with 200 and 300 mg/kg Ketamir-2 (groups 3M and 4M). Data presented in Figure 5B.

#### Time spent in center

In the settings of open field, mice tend to spend more time in the margins of the arena instead of in the center of it. There was no significant difference between time spent in the center and between treated groups. However, groups treated with Ketamir-2 displayed a trend of spending more time in the center compared to vehicle -treated mice. Ketamine did not show difference on this parameter, compared with controls. Data presented in Figure 5C.

### Elevated plus maze (EPM)

**FIGURE 6:**
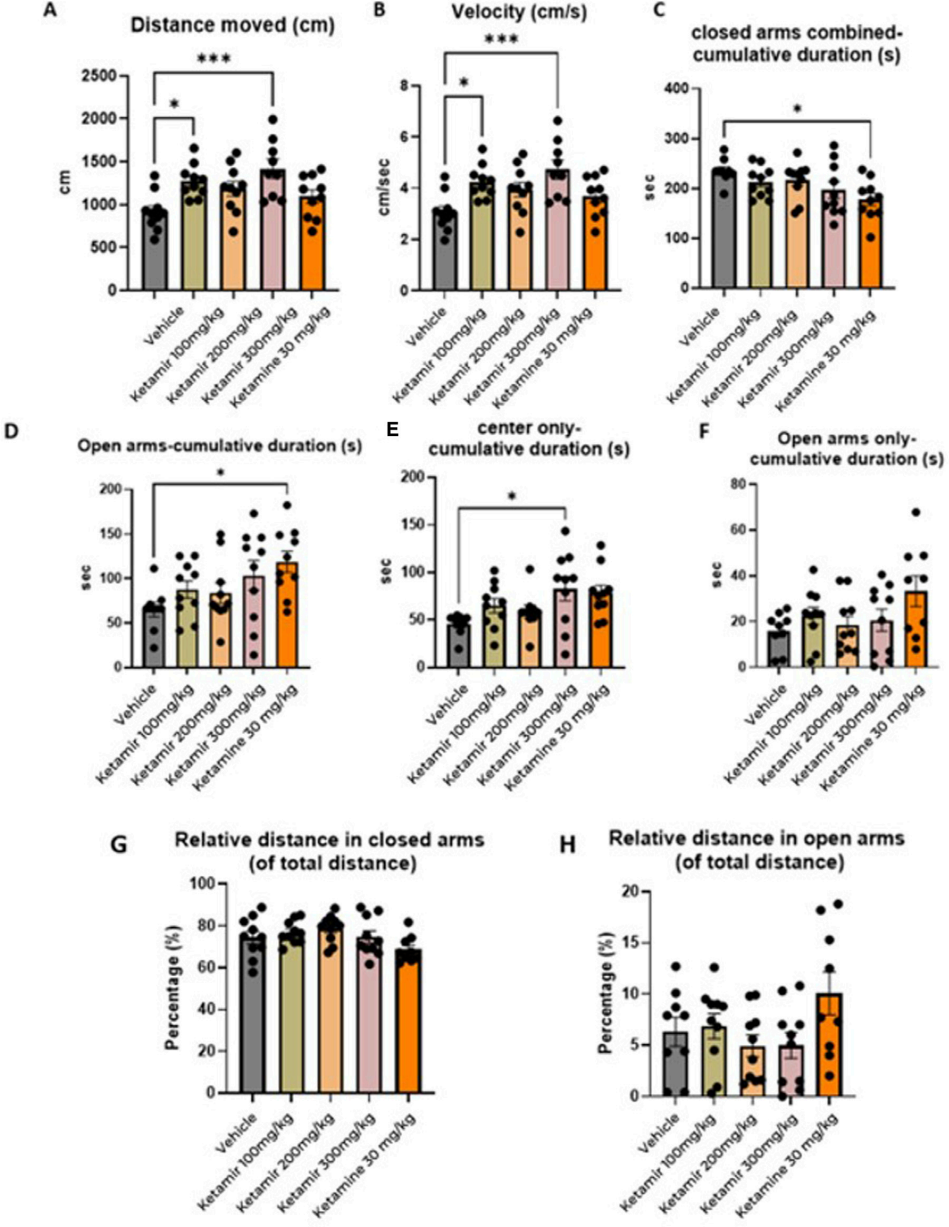
Elevated plus maze (EPM) parameters. In each group 8 mice were evaluated. **(A)** Distance moved (cm); **(B)** mean velocity (cm/seconds), **(C)** Cumulative time spend in closed arms combined (s), **(D)** cumulative duration in the open arms, **(E)** Time spent in the center, **(F)** Cumulative time spent in the open arms **(G)** relative distance in closed arms and **(H)** relative distance in open arms. One-Way ANOVA compared to vehicle control: *p < 0.05, **p < 0.01 and ***p < 0.001.

Open field was performed on Day 4 of the study, after 2 days of washout after the Open Field Test- Mice were placed in the center of a chamber and allowed to freely explore the chamber for the duration of the test session (10 min). Mice were tested and recorded using a video camera installed on the ceiling of the behavioral room and connected to a computer to record images. Behavioral parameters from the video recordings were processed and analyzed using the Ethovision software. The graphical representation of the data obtained is shown in Figure 6.

#### Distance moved

Mice treated with Vehicle exhibited significantly less travel distance during the EPM test, compared to groups treated with 100 and 300 mg/kg Ketamir-2. Data presented in Figure 6A.

#### Velocity

Mice treated with vehicle traveled slower compared to group treated with 300 mg/kg Ketamir-2. Data presented in Figure 6B.

#### Cumulative time spent in open arms

Mice treated with Ketamine spent significantly more time in the open arms, compared to vehicle. Data presented in Figure 6C.

This parameter includes both open arms and the center area between the 4 arms of the maze.

#### Cumulative time spent in the center of the maze

Mice treated with 300 mg/kg Ketamir-2 spent significantly more time in the center of the maze, compared to vehicle. Data presented in Figure 6D.

#### Cumulative time spent in open arms only (without center)

There was no significant difference between time spent in the open arms (without the area of the center) between treated groups. Data presented in Figure 6E.

### Forced swimming test (FST)

**FIGURE 7:**
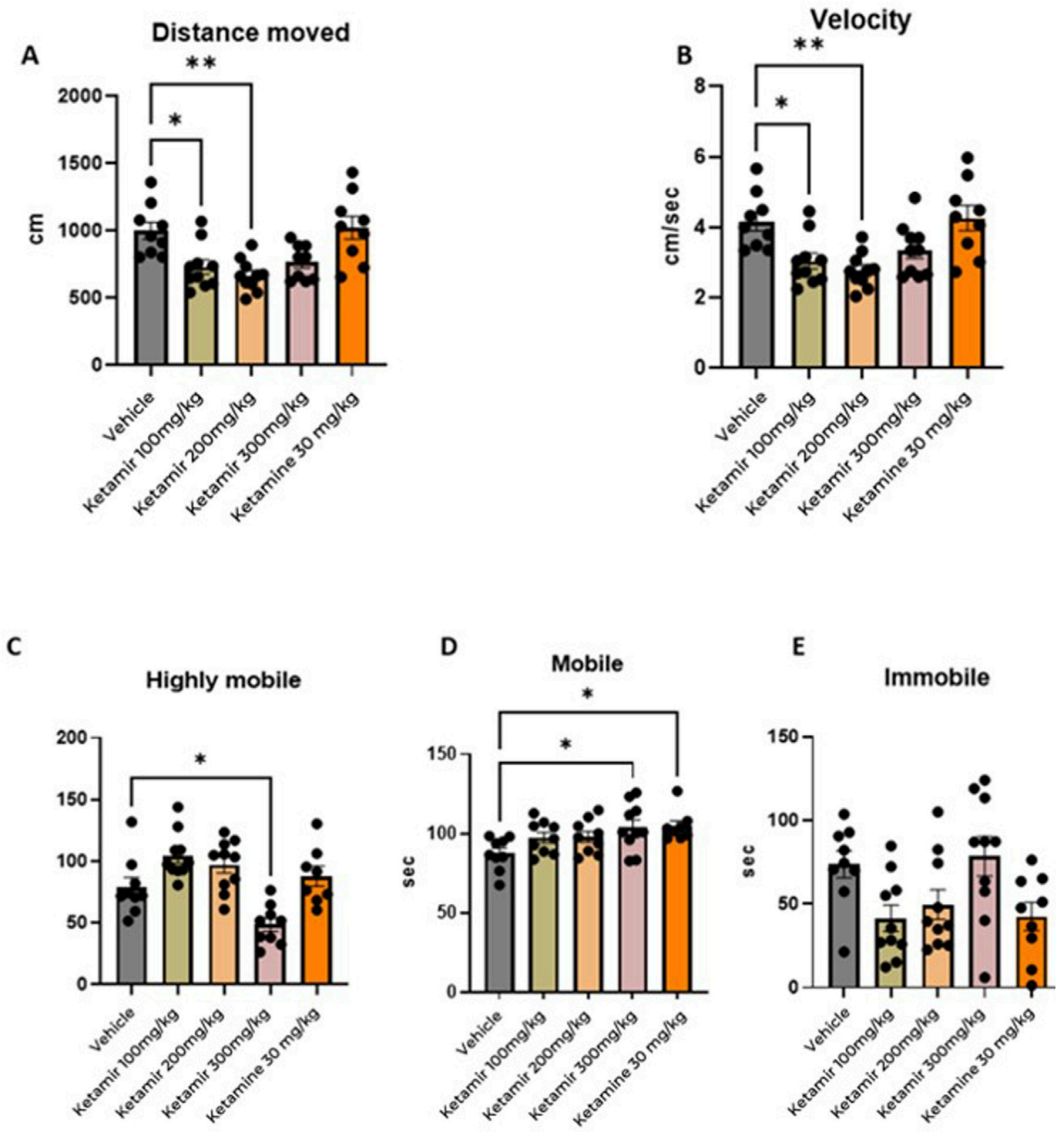
Forced swimming test (FST) parameters. In each group 8 mice were evaluated. **(A)** Distance moved (cm); **(B)** mean velocity (cm/seconds), **(C)** highly mobile (seconds), **(D)** mobile, and **(E)** immobile (seconds). One-Way ANOVA compared to vehicle control: *p < 0.05.

Forced Swim Test was performed on Day 8 of the study, after at least 2 days of washout subsequent to the EPM. Mice were placed for 6 minutes in a cylindrical filled with water. Tests were recorded using a video camera and analyzed offline. Behavioral monitoring was performed from 2 to 6 min after placing the mouse in the water. To process and analyze behavioral parameters detected in videos, the computer software Ethovision was used. The graphical representation of the data obtained is shown in Figure 7.

#### Distance moved

Mice treated with Vehicle swam significantly more distance during the FST, compared to groups treated with 100 and 200 mg/kg Ketamir. Data presented in Figure 7A.

#### Velocity

Mice treated with Vehicle (group 1M) swam faster compared to group treated with 100 and 200 mg/kg Ketamir-2 (2M and 3M respectively). Data presented in Figure 7B.

#### Mobility

Using Ethovision, analyzed the cumulative duration of each mobility state of the mouse: highly mobile/struggling, mobile/swimming and immobile/floating. Mice treated with vehicle (group 1M) were significantly longer highly mobile, meaning they displayed struggling during the test, compared to the group treated with 300 mg/kg Ketamir-2 (group 4M). (Figure 7C).

Mice treated with 300 mg/kg Ketamir-2 (group 4M) or Ketamine (group 5M) were significantly longer mobile (swimming) compared to mice from the vehicle group (group 1M).

There was no significant difference in immobility (floating state) of the mice in either of the treatments. There was some trend of less immobility in groups treated with 100 and 200 mg/kg Ketamir-2 and Ketamine (groups 2M, 3M and 5M respectively), however it is not statistically significant.

## Discussion

Ketamine’s role as an antidepressant in human and in animal models has been extensively studied, offering insights into its rapid-acting effects and mechanisms, which differ from traditional antidepressants. In preclinical research, ketamine is typically administered to rodents (mice or rats) to evaluate its ability to reverse depression-like behaviors, often in models mimicking human depressive symptoms. Common animal models include the Forced Swim test (FST) and tail suspension test (TST). In the FST and TST, animals are placed in inescapable situations (e.g., a water tank or suspended by the tail), and immobility time is measured as an indicator of behavioral despair—analogous to hopelessness in depression. Ketamine, significantly reduces immobility within 30 min to a few hours, unlike traditional antidepressants like SSRIs (e.g., fluoxetine), which require weeks to show effects. The mechanism behind this rapid action involves ketamine’s antagonism of NMDA receptors, which triggers a cascade of neurobiological changes.

Unlike its hyperlocomotion effects at higher doses, which seem to be mediated through increased Dopamine release, lower antidepressant doses (e.g., 10 mg/kg) enhance synaptic plasticity. Ketamine’s metabolite, (2R, 6R)-hydroxynorketamine (HNK), also contributes. Unlike ketamine itself, HNK lacks NMDA antagonism but still exerts antidepressant effects, possibly via AMPA receptor activation, further stimulating synaptic plasticity. In mice, HNK reduces FST immobility at doses as low as 5–10 mg/kg, suggesting it may underlie some of ketamine’s benefits without its dissociative side effects. These findings align with human studies, where ketamine (e.g., 0.5 mg/kg IV) relieves depressive symptoms within hours, paving the way for derivatives like esketamine, now FDA-approved.

While Ketamir-2 is likely binding at PCP-site of NMDA, like ketamine, it markedly differs from Ketamine in its lower affinity to this site, but also in its marked selectivity towards numerous other receptors sites and transporters that Ketamine also targets. Also, the main metabolite of Ketamir-2, unlike the metabolite of ketamine, retains low PCP binding affinity and lacks interaction with the NMDA site.

In other related studies (manuscript in preparation), Ketamir-2 given orally at similar dose ranges as in the current study, was shown to be effective in reducing pain in several models, including spinal nerve ligation, paclitaxel or diabetes induced neuropathic models (data not shown). Ketamine is widely studied for its analgesic properties, particularly in acute pain, chronic pain, and neuropathic pain. Its mechanisms involve NMDA receptor blockade, modulation of glutamatergic signaling, and interactions with other systems (e.g., opioid, monoaminergic) (Niesters et al., 2014; Hirota and Lambert, 2011).

The similarity of anti-depressant and pain reducing activities between Ketamir-2 and Ketamine, albeit the observed low affinity of Ketamir-2 and its metabolite nor-Ketamir on the NMDA PCP-site, and its lack of activity at other sites, compared with Ketamine, suggest that the PCP-site has the most important role for these specific activities.

We have a series of informational ADME assessments typical for a novel therapeutic classified as a small molecule. In model Caco-2 permeation system, it was shown that Ketamir-2 is permeable at a dose of 10 µM (A-B and B-A) and is not highly bound to plasma proteins in mouse, rat, dog, cynomolgus monkey, or human plasma (data not shown). Substrate interaction and metabolism studies indicate that Ketamir-2 is not a substrate of P-glycoprotein (data not shown). In addition, the pharmacokinetics of Ketamir-2 have been characterized in a series of *in vivo* PK/TK studies conducted in Sprague Dawley rats and Beagle dogs. General PK data indicate that Ketamir-2 does cross the blood brain barrier with longer exposure times and greater levels of nor-Ketamir present in the brain. Moreover, the PK profile of Ketamir-2 following oral administration is characterized by rapid absorption, a short half-life and high clearance (data not shown). Brain levels of the drug are expected to affect both its pharmacological activity and its selectivity towards other potential side-effects. Studies conducted in rats at high doses have recently confirmed the lack of such side effects, further confirming its good selectivity.

For Ketamine, there are evidence that ketamine is a P-gp substrate, with implications for its absorption, distribution, and efficacy in pain management (e.g., neuropathic pain) and other therapeutic contexts. The findings suggest that P-gp-mediated efflux may contribute to ketamine’s low oral bioavailability and variable central effects, which are critical for its analgesic applications (Tomkins et al., 2018). In this respect, the absence of P-gp interaction and the better oral bioavailability of Ketamir-2 (and its metabolite), compare with Ketamine, may explain that even at much lower affinity towards the PCP-site, and better selectivity, some of the pharmacological activities, such as antidepressant and pain-reducing actions can be maintained.

To further confirm the potential consequences of the lower affinity and selectivity of Ketamir-2 *in vivo*, we have evaluated the effects of Ketamir-2 administration on locomotor activity in male C57BL6/J mice. Measurements of spontaneous locomotor activity by actimetry are widely used for *in vivo* safety evaluation of new drugs on the central nervous system (CNS) in rodents.

In this study, vehicle-treated animals displayed normal time-dependent activity during the 1-h recording period. Animals treated with Ketamir-2 20 mg/kg ip and 50 mg/kg po showed a similar pattern of locomotor activity compared to the vehicle group. On the other hand, Ketamir-2 50 mg/kg ip decreased locomotor activity compared to vehicle-treated animals. Ketamine 20 mg/kg ip increased locomotor activity. The differences between the sedative effect observed upon intraperitoneal activity, compared with the lack of effect upon oral administration, may be explained by the relatively low bioavailability of ketamir-2 under these experimental conditions.

The mechanisms underlying ketamine-induced hyperlocomotion involve disruptions in glutamatergic, dopaminergic, and other neurotransmitter systems, which parallel neurochemical imbalances implicated in schizophrenia (Irifune et al., 1991)​. As studies indicate that ketamine-induced hyperlocomotion can be blocked by dopamine receptor antagonists like haloperidol, it is suggested that this behavior is mainly dopamine-linked. However, the effect is not solely dopamine-driven—ketamine’s impact on glutamate and other systems, like endocannabinoids and serotonin, suggests a broader disruption of neural networks (Xu, et al., 2020)​. The absence of effect of Ketamir-2 in this model, reflects its lack of interaction at adrenergic reuptake sites and/or dopaminergic neurons reinforcing the selectivity of Ketamir-2 for the NMDA receptor.

The Open Field Test (OFT), Elevated Plus Maze (EPM), and Forced Swimming Test (FST) are commonly employed behavioral assays used to evaluate the efficacy of anxiety and depression medications. In the Open Field Test (OFT), mice treated with antidepressants typically exhibit greater locomotor activity, moving more extensively and exploring a larger area of the open field compared to control mice. These treated mice are also more inclined to spend time in the central region of the open field, which is usually avoided by anxious animals. Such behavior indicates a decrease in anxiety and an increase in exploratory tendencies. According to the results presented in Figure 5, mice treated with all tested concentrations of Ketamir-2 moved larger distance than vehicle treated mice. Mice treated with 100 and 200 mg/kg Ketamir-2 also were significantly faster, with higher velocity compared to control. Mice treated with Ketamir-2 also showed a trend of more time spent in the center, however it was not statistically significant.

In the Elevated Plus Maze (EPM), mice treated with antidepressants typically demonstrate increased exploratory behavior and reduced anxiety. These mice are more likely to enter and spend more time in the open arms of the maze, areas that anxious animals usually avoid. This increased presence in the open arms suggests an anxiolytic effect and enhanced willingness to explore novel environments. According to the results presented in Figure 6, mice treated with 100 or 300 mg/kg Ketamir-2 moved larger distance than vehicle treated mice. Mice treated with 300 mg/kg Ketamir-2 also significantly were faster, with higher velocity, and spent more time in the center of the maze, compared to control. However, there was no significant difference in time spent in the open arms only (without the center zone) of Ketamir-2 treated mice compared with control. Ketamine treated mice did spend significantly more time in the open arms, in the center is included.

In the Forced Swimming Test (FST), mice treated with antidepressants are generally more mobile. Antidepressant-treated mice show reduced immobility time compared to control mice. Instead of remaining immobile, they exhibit increased active behaviors such as swimming and climbing. This increased activity is interpreted as a sign of decreased behavioral despair and an antidepressant-like effect. According to the results presented in Figure 7, mice treated with Ketamir-2 show reduction in traveled distance and lower velocity (300 mg/kg Ketamir concentration shows similar trend to the rest of the concentrations, however it is not statistically significant). When mobility state was analyzed, 300 mg/kg of Ketamir-2 is the only Ketamir-2 treatment that reduced hyper-mobility, and increased mobility significantly. No treatment significantly induced immobility compared to vehicle treated, however-100 and 200 mg/kg Ketamir-2 and Ketamine showed a trend of reduced immobility.

Ketamine was used as a positive control in this experiment however, in many parameters it did not differ from vehicle treatment, or its response was opposite to that of Ketamir-2. The lasting antidepressant effects of ketamine may vary depending on the treatment regimen, concentration, and the time studied after administration. Therefore, these parameters should be carefully examined in future studies.

## Materials and methods

### Animals

Animal handling was performed according to the guidelines of the National Institute of Health (NIH) and the Association for Assessment and Accreditation of Laboratory Animal Care (AAALAC) or accredited facilities under EU and French animal welfare regulations for animal use in experimentation (European Directive 2010/63/EU and French decrees and orders 2013-118 of 1 February 2013, and 2020-274 of 17 March 2020). The hyperlocomotion study was approved by the Biotrial Ethics Committee “Comité de Réflexion Ethique en Expérimentation Animale (CR2EA) (registered by the “Ministère de l’Enseignement Supérieur et de la Recherche et de l’Innovation” (French ministry of higher education, research, and innovation).

The protocols of the antidepressant/anxiolytic studies were approved under “The Israel Animal Welfare Act” and following “The Israel Board for Animal Experiments” Ethics Committee approval. All efforts were made to minimize animal discomfort by use of anesthesia/analgesia. This study was performed in compliance with “The Israel Animal Welfare Act” and following “The Israel Board for Animal Experiments” Ethics Committee approval # NPC-Ph - IL - 2,404–264.

For the locomotor activity study, 2-months old Male C57BL6/J mice (Janvier Labs, Saint-Berthevin, France) were used.

For the other behavioral tests, 2–2.5 months old Male C57BL/6 J mice (Envigo RMS Ltd., Israel) were used.

All animals were maintained in 12-hour-light/12-hour-dark conditions in groups of up to 10 in polysulfone cages (for the locomotor activity study) and in individually ventilated cages (for OFT, EPM, FST) with *ad libitum* access to food and water. Every effort was made to reduce the number of mice used and minimize their suffering.

### Behavioral tests

All behavioral tests were done with a single administration and were performed 20 min post-test, item administration unless otherwise indicated. All behavioral assessments were conducted by experimenters blinded to the treatment groups.

### Spontaneous locomotor activity

Spontaneous locomotor activity (measurement of horizontal activity counts) was recorded over a period of 1 h in 5-min intervals, starting just after administration. An activity count will be recorded each time the mouse interrupts one of the infrared cell beams.

All parameters were analyzed using GraphPad Prism^®^ software. Statistical tests were performed with an alpha level of 0.05, except for interaction test, for which the alpha level is 0.10. Tables presenting individual data as well as descriptive statistics (n, mean, SD, SEM, Min, median and max) by group and graphs for each group will be provided. The following analyses were performed for measurement times 0-5, 5-10, 10-15, 15-20, 20-25, 25-30, 30-35, 35-40, 40-45, 45-50, 50-55, 55–60 min.

### Open field test

Open field was performed on Day 1 of the study. Mice had a single oral administration of the drug or vehicle and were performed 20 min post-test. They were placed in the center of a chamber and allowed to freely explore the chamber for the duration of the test session (10 min). Mice were tested and recorded using a video camera installed on the ceiling of the behavioral room and connected to a computer to record images. To process and analyze behavioral parameters detected in videos, the computer software Ethovision was used.

### Elevated plus maze

The Elevated Plus Maze is generally used for the assessment of anxiety-related behavior. A plus-shaped maze containing two dark and enclosed arms and two open and lit arms, elevated 100 cm above ground, was used. The arms were 30 × 5 cm with a 5 × 5 cm center area, and the walls of the closed arms were 40 cm high. Animals were acclimated to the testing room for at least 30 min prior to the testing. Mice had a single oral administration of the drug or vehicle and test performed 20 min post-test. Mice were placed in the center of a chamber and allowed to freely explore the chamber for the duration of the test session (5 min). Mice were tested and recorded using a video camera installed on the ceiling of the behavioral room and connected to a computer to record images. To process and analyze behavioral parameters detected in videos, the computer software Ethovision was used.

### Forced swim test

Forced Swim Test was performed on Day 8 of the study, after at least 2 days of washout after the EPM. Mice had a single oral administration of the drug or vehicle and test performed 20 min post-test. Mice were placed for 6 minutes in a cylindrical filled with water. Animals were acclimated to the testing room for at least 15 min prior to the testing, then mice were placed for 6 minutes in a cylindrical Plexiglas container (around 20 cm diameter) filled with water at 22°C–25°C and a depth of at least 20 cm (same depth should be used for all mice). Tests were recorded using a video camera and analyzed offline. Behavioral monitoring was performed from 2 to 6 min after placing the mouse in the water. To process and analyze behavioral parameters detected in videos, the computer software Ethovision was used.

### Drugs and reagents

The test item was synthetized by Recipharm. *In vitro* studies were carried out using the Ketamir-2 HCl salt. For *in vivo* studies, Ketamir-2 pamoate was used. It was suspended in 5% DMSO, followed by 40% HPβCD (in water). The mixture was subjected to alternating cycles of sonication and vortexing over several hours until a homogeneous suspension was achieved. Stock was then aliquoted to tubes and kept frozen at −20°C refrigerator until used. Before each administration, test item was thawed, sonicated and diluted to final concentration per treatment group. Ketamine was provided by VetVive.

## Data Availability

The original contributions presented in the study are included in the article/Supplementary Material, further inquiries can be directed to the corresponding author.

## Abbreviations

AMPA, α-Amino-3-hydroxy-5-methyl-4-isoxazolepropionic Acid; ANOVA, Analysis of Variance; DMSO, Dimethyl sulfoxide; EPM, Elevated plus maze; FST, Forced swim test; GPCR, G-protein coupled receptors; HP-β-CD, 2-Hydroxypropyl-β-cyclodextrin; HNK, (2R,6R)-hydroxynorketamine; HPE, High-Point Effect; ND, Not determined; NET, Norepinephrine transporter; NMDA, N-methyl-D-aspartate; PK, Pharmacokinetics; PO, Per os (by mouth); SEM, Standard Error of the Mean; SERT, Serotonin Transporter; Std Dev, Standard deviation; SOPs, Standard operating procedures; TK, Toxicokinetics; TST, Tail suspension test; ZPE, Zero-point Effect.
