# Supplementary material for: KETAMIR-2, a new molecular entity and novel ketamine analog
Source: Front Pharmacol. 2025 Jun 20;16:1606976. doi: 10.3389/fphar.2025.1606976 (PMC12231361; doi:10.3389/fphar.2025.1606976)
Supplement: Supplementary file 1 [file DataSheet1.pdf]

Supplement:

In vitro methods.

Study of Ketamir-2 HCl

Assay	Source	Ligand	Conc.	Kd	Non Specific	Incubation	Detection Method
**Ion channels**							
**Glutamate (Non-Selective) Rat Ion Channel Glycine (Strychnine-Insensitive),Mass Spectrometry Binding**	rat brain	MDL 105.519	2 nM	1.7 nM	5,7 dichlorokynurenic acid (10 µM)	60 min à 0°C	MS
**AMPA (agonist radioligand)**	rat cerebral cortex	[^{3}H]AMPA	8 nM	82 nM	L-glutamate (1 mM)	60 min 4°C	Scintillation counting
**kainate (agonist radioligand)**	rat cerebral cortex	[^{3}H]kainic acid	5 nM	19 nM	L-glutamate (1 mM)	60 min 4°C	Scintillation counting
**NMDA (antagonist radioligand)**	rat cerebral cortex	[^{3}H]CGP 39653	5 nM	23 nM	L-glutamate (100 µM)	60 min 4°C	Scintillation counting
**PCP (antagonist radioligand)**	rat cerebral cortex	[^{3}H]TCP	10 nM	13 nM	MK 801 (10 µM)	120 min 37°C	Scintillation counting

Study of nor-Ketamir

Assay	Source	Ligand	Conc.	Kd	Non Specific	Incubation	Detection Method
**Ion channels**							
**NMDA (antagonist radioligand)**	rat cerebral cortex	[^{3}H]CGP 39653	5 nM	23 nM	L-glutamate (100 µM)	60 min 4°C	Scintillation counting
**PCP (antagonist radioligand)**	rat cerebral cortex	[^{3}H]TCP	10 nM	13 nM	MK 801 (10 µM)	120 min 37°C	Scintillation counting

Selectivity In Vitro Studies

Ion Channels

The cell lines grew in a humidified incubator at 37°C under 5% carbon dioxide.

Cell	Culture medium
HEK293/ hERG	DMEM + 10% Fetal bovine serum +0.8 mg/mL G418

CHOK1/ hKv7.1	F12K + 10% Fetal bovine serum + 6 µg/mL Puromycin + 0.8 mg/mL G418
HEK293/ HTR3A	DMEM + 10% Fetal bovine serum +6 µg/mL Puromycin + 0.8 mg/mL G418
CHOK1/ GABA	F12K + 10% Fetal bovine serum + 6 µg/mL Puromycin + 0.8 mg/mL G418+300 µg/mL Hygromycin
CHOK1/ nAchR	F12K + 10% Fetal bovine serum + 6 µg/mL Puromycin + 0.8 mg/mL G418

The voltage-clamp pulse protocol is performed to record ion channel using whole cell patch clamp technology. hERG current is recorded under a holding potential at -80 mV and then depolarize to 30 mV for 2.5 seconds to activate the hERG channel. The peak tail current is induced by a repolarizing pulse to -50 mV for 4 seconds. hKv7.1 current is recorded under a holding potential at -80 mV and the current is induced by a pulse to +30 mV for 2 seconds. HTR3A, GABA and nAchR currents are recorded under a holding potential at -80 mV. Cells are incubated with compound for 5 minutes, or until the current reach a steady-state level. All tests are performed at room temperature.

In vitro effect of Ketamir on the five ion channels current

**Antagonist**						
	HPE			Ketamir 10 µM		
	%Inhibition1	%Inhibition2	Mean	%Inhibition1	%Inhibition2	Mean
hERG	98.47	97.13	97.80	1.06	9.41	5.24
hKv7.1	90.02	91.13	90.58	21.73	17.88	19.80
HTR3A	94.33	91.87	93.10	15.84	7.11	11.47
GABA	94.33	93.87	94.10	11.91	24.91	18.41
nAchR	87.09	81.32	84.21	5.82	16.62	11.22
**Agonist**						

	HPE			Ketamir 10 μM		
	%Effect1	%Effect2	Mean	%Effect1	%Effect2	Mean
HTR3A	100.00	100.00	100.00	0.00	0.00	0.00
GABA	100.00	100.00	100.00	0.00	0.00	0.00
nAchR	100.00	100.00	100.00	0.00	0.00	0.00

GPCR

The cell lines grew in a humidified incubator at 37°C under 5% carbon dioxide. For cAMP assay the following conditions were used:

**Cell**	**Culture medium**
CHOK1/A2A	F12K + 10% Fetal bovine serum + 10 μg/mL Puromycin
CHOK1/beta1	F12K + 10% Fetal bovine serum + 6 μg/mL Puromycin
CHOK1/beta2	F12K + 10% Fetal bovine serum + 10 μg/mL Puromycin
CHOK1/CB1	F12K + 10% Fetal bovine serum + 6 μg/mL Puromycin
CHOK1/CB2	F12K + 10% Fetal bovine serum + 6 μg/mL Puromycin
HEK293/D1	DMEM + 10% Fetal bovine serum + 3 μg/mL Puromycin
CHOK1/D2	F12K + 10% Fetal bovine serum + 10 μg/mL Puromycin

CHOK1/H2	F12K + 10% Fetal bovine serum + 6 µg/mL Puromycin
CHOK1/M2	F12K + 10% Fetal bovine serum + 6 µg/mL Puromycin
CHO/delta receptor	F12K + 10% Fetal bovine serum + 100 µg/mL Hygromycin
CHO/kappa receptor	F12K + 10% Fetal bovine serum + 100 µg/mL Hygromycin
CHO/Mu receptor	F12K + 10% Fetal bovine serum + 100 µg/mL Hygromycin
CHOK1/5-HT1A	F12K + 10% Fetal bovine serum + 6 µg/mL Puromycin
CHOK1/5-HT1B	F12K + 10% Fetal bovine serum + 6 µg/mL Puromycin

For IP1 Assay cell lines grew in a humidified incubator at 37°C under 5% carbon dioxide using the following conditions.

Cell	Culture medium
CHOK1/M3	F12K + 10% Fetal bovine serum + 6 µg/mL Puromycin
CHOK1/alpha1A	F12K + 10% Fetal bovine serum + 6 µg/mL Puromycin
CHOK1/CCKA	F12K + 10% Fetal bovine serum + 8 µg/mL Puromycin
CHO/H1	DMEM + 10% Fetal bovine serum + 8 µg/mL Puromycin
CHOK1/M1	F12K + 10% Fetal bovine serum + 4 µg/mL Puromycin
CHOK1/V1A	F12K + 10% Fetal bovine serum + 6 µg/mL Puromycin
CHO/ETA	DMEM + 10% Fetal bovine serum + 8 µg/mL Puromycin

CHOK1/5-HT2A	F12K + 10% Fetal bovine serum + 6 µg/mL Puromycin
CHOK1/5-HT2B	F12K + 10% Fetal bovine serum + 6 µg/mL Puromycin

Summary of %effect and %inhibition of compounds

Agonist									
	ZPE			HPE			Ketamir 10µM		
Target	%Effect1	%Effect2	Mean	%Effect1	%Effect2	Mean	%Effect1	%Effect2	Mean
CB1/CNR1	-2.33	2.33	0.00	96.82	103.18	100.00	15.05	-7.77	3.64
M2/CHRM 2	5.37	-5.37	0.00	97.89	102.11	100.00	-7.56	-22.95	-15.26
delta/OPR D1	5.02	-5.02	0.00	97.61	102.39	100.00	7.21	6.15	6.68
kappa/OPR K1	4.54	-4.54	0.00	98.68	101.32	100.00	-5.26	-1.51	-3.38
mu/OPRM 1	-3.16	3.16	0.00	99.29	100.71	100.00	-0.53	-3.77	-2.15
5-HT1A/HT R1A	0.98	-0.98	0.00	101.02	98.98	100.00	-2.73	1.04	-0.84
5-HT1B/HT R1B	-1.60	1.60	0.00	97.47	102.53	100.00	-5.01	1.40	-1.80
M3/CHRM 3	0.03	-0.03	0.00	95.98	104.02	100.00	-0.39	0.37	-0.01
alpha1A/A DRA1A	0.36	-0.36	0.00	95.77	104.23	100.00	1.56	1.23	1.39
CCKA/CC KAR	-0.37	0.37	0.00	96.33	103.67	100.00	0.60	0.51	0.56
H1/HRH1	0.59	-0.59	0.00	103.55	96.45	100.00	0.03	1.44	0.74

M1/CHRM 1	0.11	-0.11	0.00	96.63	103.37	100.00	-0.84	-0.27	-0.56
V1A/AVP R1A	-0.95	0.95	0.00	106.82	93.18	100.00	-0.44	1.61	0.58
A2A/ADO RA2A	-0.22	0.22	0.00	100.82	99.18	100.00	0.02	0.58	0.30
beta1/ADR B1	0.03	-0.03	0.00	101.33	98.67	100.00	0.19	0.25	0.22
D2/DRD2	2.30	-2.30	0.00	101.58	98.42	100.00	-4.66	-12.46	-8.56
H2/HRH2	-0.05	0.05	0.00	100.50	99.50	100.00	0.47	0.28	0.37
CB2/CNR2	-10.02	10.02	0.00	100.40	99.60	100.00	15.34	-0.87	7.24
D1/DRD1	-0.02	0.02	0.00	109.38	90.62	100.00	0.68	0.27	0.47
beta2/ADR B2	0.02	-0.02	0.00	95.05	104.95	100.00	0.36	0.22	0.29
ETA/EDN RA	4.05	-4.05	0.00	81.29	118.71	100.00	4.65	25.02	14.84
5-HT2A/HT R2A	-0.17	0.17	0.00	104.60	95.40	100.00	-1.67	-0.53	-1.10
5-HT2B/HT R2B	-2.33	2.33	0.00	100.17	99.83	100.00	-0.43	2.13	0.85

**Antagonist**									
	ZPE			HPE			Ketamir 10μM		
Target	%Inhibitio n1	%Inhibitio n2	Mean	%Inhibitio n1	%Inhibitio n2	Mean	%Inhibitio n1	%Inhibition 2	Mean
CB1/CNR1	-1.56	1.56	0.00	102.30	97.70	100.00	4.93	7.27	6.10
delta/OPR D1	-0.56	0.56	0.00	90.90	109.10	100.00	6.28	5.03	5.66
5-HT1A/HT R1A	7.02	-7.02	0.00	101.51	98.49	100.00	9.00	16.22	12.61

M2/CHRM 2	-3.52	3.52	0.00	104.79	95.21	100.00	-5.96	6.31	0.17
mu/OPRM 1	0.27	-0.27	0.00	101.97	98.03	100.00	0.06	2.61	1.33
5-HT1B/HT R1B	-3.05	3.05	0.00	98.06	101.94	100.00	-8.10	5.46	-1.32
M3/CHRM 3	-0.37	0.37	0.00	100.21	99.79	100.00	14.96	1.72	8.34
alpha1A/A DRA1A	-3.62	3.62	0.00	99.87	100.13	100.00	9.84	8.18	9.01
CCKA/CC KAR	-2.10	2.10	0.00	100.18	99.82	100.00	5.64	9.16	7.40
H1/HRH1	1.43	-1.43	0.00	100.53	99.47	100.00	3.71	-6.79	-1.54
M1/CHRM 1	-5.35	5.35	0.00	100.34	99.66	100.00	-8.74	-7.11	-7.93
V1A/AVP R1A	6.23	-6.23	0.00	99.34	100.66	100.00	-3.73	-11.66	-7.69
A2A/ADO RA2A	0.23	-0.23	0.00	99.85	100.15	100.00	-10.46	-7.47	-8.97
beta1/ADR B1	-1.87	1.87	0.00	100.02	99.98	100.00	-2.85	-8.56	-5.71
D2/DRD2	0.63	-0.63	0.00	96.16	103.84	100.00	-1.09	2.02	0.46
H2/HRH2	-0.81	0.81	0.00	99.86	100.14	100.00	-1.94	11.10	4.58
CB2/CNR2	-3.52	3.52	0.00	100.41	99.59	100.00	1.20	12.40	6.80
kappa/OPR K1	4.86	-4.86	0.00	101.25	98.75	100.00	6.68	4.44	5.56
D1/DRD1	1.26	-1.26	0.00	100.21	99.79	100.00	-4.07	-8.79	-6.43
beta2/ADR B2	-2.96	2.96	0.00	100.05	99.95	100.00	5.74	-2.73	1.51
ETA/EDN RA	2.32	-2.32	0.00	88.28	111.72	100.00	-24.81	-8.26	-16.53

5-HT2A/HTR2A	2.40	-2.40	0.00	99.32	100.68	100.00	-7.30	-0.85	-4.07
5-HT2B/HTR2B	-3.57	3.57	0.00	102.47	97.53	100.00	11.38	5.73	8.56
